# Structural elucidation of substrate-bound aminoglycoside acetyltransferase (3)-IIIa

**DOI:** 10.1371/journal.pone.0269684

**Published:** 2022-08-03

**Authors:** Michał Zieliński, Jonathan Blanchet, Sophia Hailemariam, Albert M. Berghuis

**Affiliations:** 1 Department of Biochemistry, McGill University, Montréal, Québec, Canada; 2 Centre de Recherche en Biologie Structurale, McGill University, Montréal, Québec, Canada; 3 Department of Microbiology and Immunology, McGill University, Montréal, Québec, Canada; 4 McGill Antimicrobial Resistance Centre, McGill University, Montréal, Québec, Canada; Laurentian University, CANADA

## Abstract

Canonical aminoglycosides are a large group of antibiotics, where the part of chemical diversity stems from the substitution of the neamine ring system on positions 5 and 6. Certain aminoglycoside modifying enzymes can modify a broad range of 4,5- and 4,6-disubstituted aminoglycosides, with some as many as 15. This study presents the structural and kinetic results describing a promiscuous aminoglycoside acetyltransferase AAC(3)-IIIa. This enzyme has been crystallized in ternary complex with coenzyme A and 4,5- and 4,6-disubstituted aminoglycosides. We have followed up this work with kinetic characterization utilizing a panel of diverse aminoglycosides, including a next-generation aminoglycoside, plazomicin. Lastly, we observed an alternative binding mode of gentamicin in the aminoglycoside binding site, which was proven to be a crystallographic artifact based on mutagenesis.

## Introduction

The field of medicine was revolutionized with the discovery of antibiotics. Infections that were previously deemed as critical became treatable with a course of antimicrobial chemotherapy. Aminoglycosides were amongst one of the first classes of antibiotics discovered, with streptomycin being the earliest member of this group employed in the clinical setting [1]. This family of antibiotics possess broad-spectrum antibacterial action against bacterial pathogens, including *Brucellae*, *Corynebacterium jeikeium*, *Pseudomonas aeruginosa*, *Listeriae* and *Francisella tularensis* [2]. Moreover, their synergistic action in combination with other antibiotics has enhanced the utility of these agents [3]. However, the sustained and frivolous use of aminoglycosides in the clinic and agriculture has encouraged the selection and spread of genetic elements encoding for aminoglycoside resistance, where aminoglycoside modifying enzymes (AGMEs) confer the highest levels of resistance to this antibiotic class [4].

There are three classes of AGMEs that can detoxify aminoglycosides through either acetylation, phosphorylation or nucleotidylation [4]. Aminoglycoside acetyltransferases (AACs) are a member of the Gcn5-related N-acetyltransferases (GNAT) superfamily of proteins that span all domains of life; representing over 10,000 enzymes; where they utilize acetyl-CoA to modify their cognate substrate [5]. In the context of aminoglycoside resistance, AAC enzymes are of great medical interest as they are spread across the clinical resistome [4]. Although it is not uncommon for aminoglycoside modifying enzymes to exhibit selectivity for either 4,5- and 4,6-disubstituted aminoglycosides, AAC(3)-III isozymes are able to utilize them both [6]. In fact, AAC(3)-III isozymes are the most promiscuous aminoglycoside acetyltransferases capable of utilizing: tobramycin, gentamicin, dibekacin, sisomicin, kanamycin, netilmicin, paromomycin and lividomycin [7]. Specifically, AAC(3)-IIIa and its fellow isozymes catalyze the transfer of an acetyl group of acetyl-CoA to the 3 position of the aminoglycoside’s canonical 2-deoxystreptamine (DOS) ring [6].

Here, we present three crystal structures of AAC(3)-IIIa isozyme (iso^A^) from *Pseudomonas aeruginosa* in complex with 4,5- disubstituted neomycin and 4,6-disubstituted sisomicin and gentamicin. These structures, in conjunction with kinetic experiments, have allowed us to dissect iso^A^’s ability to bind and modify a wide range of aminoglycosides from two chemically unique groups. Here, we outline the structural and kinetic basis of iso^A^’s ability to utilize its tripartite active site to render many clinically useful antibiotics ineffective.

## Materials and methods

### AAC(3)-IIIa gene synthesis

The AAC(3)-IIIa gene from *Pseudomonas aeruginosa* (GenBank: CAA39184.1) was synthesized by BioBasic Inc. with codons optimized for expression in *E*. *coli*. The sequence was then subcloned into the pET15b expression vector between the *NdeI* and *BamHI* restriction sites, resulting in a hexahistidine tag and a thrombin cleavage site at the N-terminus. The DNA sequence was verified by the McGill University and Genome Quebec Innovation Centre (MUGQIC). The resulting vector was used to transform LOBSTR *E*. *coli* cells.

### Generation of AAC(3)-IIIa mutants

AAC(3)-IIIa D72W, E123F, Y64F, and Y146F mutants were generated by site-directed mutagenesis. Primers containing the modified sequences were synthesized by BioCorp DNA Inc (Montreal) (Table 1). The PCR reaction mixture was prepared using 6 μM of primers, 150 ng of template DNA, 0.5 mM of each deoxynucleoside triphosphate (dNTP) and PfuX7 enzyme [8]. The reaction was run for 20 cycles of: 30 seconds denaturation (95°C), 1 minute of annealing (44°C), and 16 minutes elongation (68°C). The presence of PCR product was verified on an agarose gel.

**Table 1:** Primers used to generate AAC(3)-IIIa mutants.

Mutant	Primers
D72W	F: 5’-GCCGGCTGGCAGGACATCCCATGGTTCATC R: 5’-CGTCCGGCAGAGAGTCGATGAACCATGGGAT
E123F	F: 5’-CGTTCACCGTTCTGCGAACCCGTTCGCGTC R: 5’-CGACCAACAGCAACCATGGACGCGAACGGGT
Y64F	F: 5’-CGGACGGTACCCTGATGATGTTCGCCGGC R: 5’-CCGGGATGTCCTGCCAGCCGGCGAACATC
Y146F	F: 5’-TAACCACGCGCTGGACTACGGTTTCGGTGTTG R: 5’-GTTTCGCTAGCGGAGATTCAACACCGAAACCGT

### Protein expression and purification

Protein expression of all constructs was carried out according to the Studier method for autoinduction [9]. Briefly, a 1 mL noninducing starter culture grown in ZYP-0.8G for 4 hours at 37˚C was used to inoculate 1 L of ZYP-5052 autoinducing media containing 100 mg mL^-1^ ampicillin. The culture was incubated at 37˚C for 3 hours, followed by 20˚C for 16 hours at 220 rpm. Cells were harvested by centrifugation at 7000 x g for 15 minutes and resuspended in 70 mL of equilibration buffer containing 50 mM Tris pH 8.0, 300 mM NaCl, and 20 mM imidazole. Cells were lysed by sonication, and debris was removed by centrifugation at 40000 x g for 20 minutes at 4˚C. The supernatant was further clarified through a 0.45 μm syringe-driven filter.

The resulting filtrate was injected on a 5 mL Ni-NTA Superflow cartridge (Qiagen) equilibrated in the aforementioned buffer and eluted with a 0–300 mM imidazole gradient over 15 column volumes. The elution fractions containing the protein were pooled then concentrated and buffer exchanged with a Vivaspin 10,000 MWCO, PES spin concentrator into a storage buffer containing 20 mM Tris pH 7.5 and 150 mM NaCl.

The hexahistidine tag was removed by thrombin digestion, where AAC(3)-IIIa was treated with 50 units of thrombin at 4°C for 16h. The digested solution was applied on a Ni-NTA cartridge followed by a HiTrap Benzamidine FF (GE) equilibrated in the storage buffer to remove the remaining His-Tagged protein and thrombin, respectively. AAC(3)-IIIa fractions were subsequently applied to HiLoad Superdex 200 26/60 column (GE) equilibrated in the storage buffer. Elution analysis showed that AAC(3)-IIIa elutes as a dimer (~60 kDa). The protein was concentrated to 15 mg ml^-1^ and stored at 4˚C.

### Protein crystallization

All optimized crystals of AAC(3)-IIIa complexes were grown at 22˚C using the sitting-drop vapour diffusion method. Drops contained at 3:2 ratio of 10 mg mL^-1^ AAC(3)-IIIa in storage buffer supplemented with 3 mM coenzyme A (CoASH) and 6 mM antibiotic. Crystals of AAC(3)-IIIa in complex with gentamicin and CoASH were obtained when the reservoir solution consisted of 0.2 M LiSO_4_, 0.1 M sodium acetate, 0.1 HEPES pH 7.5, 3% 1,3-propanediol, and 24% PEG 4000. Crystals of AAC(3)-IIIa in complex with sisomicin and CoASH were obtained when the reservoir solution consisted on 0.2 M KSCN, 6% 1,3-propanediol, and 19% PEG 3350.

Crystals of AAC(3)-IIIa in complex with neomycin and CoASH were optimized using the microseed matrix screening (MMS) method. The initial crystals used for seed stock preparation were obtained when drops contained a 1:1 ratio of 10 mg mL^-1^ AAC(3)-IIIa in storage buffer supplemented with 3 mM CoASH and 6 mM antibiotic, and reservoir solution that consisted of 0.194 M NaI, 19.4% PEG 3350, and 3% 1,3-propanediol. A 2-fold serial dilution of the seed stock was prepared using the above-mentioned buffer. Final crystals of AAC(3)-IIIa in complex with neomycin and CoASH grew when drops contained a 3:2:1 ratio of 10 mg mL^-1^ protein solution: reservoir solution: diluted seed stock, where the reservoir solution consisted of 0.2 M KSCN, 2% 1,3-propanediol, and 21% PEG 3350.

Prior to data collection, all crystals were cryo-protected using a soaking mixture composed of their respective crystallization solutions supplemented with 20% glycerol.

### Data collection and structure determination

Diffraction data for all the crystals were collected on a Bruker D8 Venture MetalJet home source detector mounted on a KAPPA goniometer. Datasets for all structures were processed using the PROTEUM3 suite (version 2018.7–2). AAC(3)-IIIa in complex with CoASH and gentamicin was solved by molecular replacement (MR) using *PHASER* [10], with an AAC(3) homologue in complex with CoASH (PDB ID: 5HT0), stripped of all non-protein atoms as the search model (version 2.8.3). AAC(3)-IIIa structures in complex with CoASH and either sisomicin or neomycin were solved using the previously mentioned gentamicin complex as a search model, stripped of all non-protein atoms. The R_free_ flags were kept consistent with all three structures to reduce the crystallographic bias.

All three structures were refined by iterative cycles of reciprocal-space refinement with *phenix*.*refine* and real-space refinement and model building in *Coot* [11,12]. AAC(3)-IIIa structural models with gentamicin, sisomicin and neomycin were deposited in the PDB (PDB IDs: 7MQM, 7MQK, and 7MQL, respectively). The final data collection and refinement statistics are summarized in Table 2.

**Table 2:** Data collection and refinement statistics.

	AAC(3)-IIIa + CoASH + Gentamicin	AAC(3)-IIIa + CoASH + Sisomicin	AAC(3)-IIIa + CoASH + Neomycin
PDB ID	7MQM	7MQK	7MQL
Data collection statistics			
Resolution range (Å)	31.05–1.6 (1.657–1.6)	32.54–1.6 (1.657–1.6)	31.05–1.6 (1.657–1.6)
Space group	P 21 21 2	P 21 21 2	P 21 21 2
Unit cell (Å, °)	130.6 91.1 100.5	130.2 90.9 100.0	130.6 90.9 100.4
Total reflections	315897 (31178)	313096 (30990)	313088 (29292)
Unique reflections	157949 (15589)	156548 (15495)	156544 (15589)
Multiplicity	24.0 (13.9)	23.4 (10.9)	13.7 (4.9)
Completeness (%)	99.4 (99.7)	99.9 (100.0)	99.8 (99.2)
Mean I/ σ(I)	42.3 (6.04)	20.9 (4.25)	20.9 (4.2)
Wilson B-factor (Å^2^)	8.7	9.1	12.9
R-merge	0.051 (0.473)	0.031 (0.259)	0.031 (0.259)
R-meas	0.073 (0.669)	0.044 (0.366)	0.044 (0.367)
CC1/2	1.00 (0.21)	1.00 (0.61)	1.00 (0.60)
CC*	1.00 (0.59)	1.00 (0.87)	1.00 (0.87)
Refinement statistics			
R-work	0.19 (0.37)	0.18 (0.31)	0.18 (0.34)
R-free^a^	0.22 (0.39)	0.20 (0.31)	0.20 (0.36)
Number of non-hydrogen atoms	9911	9825	10178
macromolecules	8178	8169	8350
ligands	417	322	532
solvent	1316	1334	1296
Protein residues	1043	1042	1043
RMS(bonds, (Å^2^))	0.007	0.013	0.008
RMS(angles, (°))	0.93	1.15	1.06
Ramachandran favored (%)	96.9	97.4	97.3
Ramachandran outliers (%)	0.0	0.1	0.1
Clashscore	2.62	2.09	2.95
Average B-factor	17.9	15.4	21.1
macromolecules	15.8	13.3	19.2
ligands	19.2	17.7	26.4
solvent	30.5	28.1	31.3

Statistics for the highest-resolution shell are shown in parentheses.

^a^ R_free_ was calculated by randomly omitting 10% of observed reflections from refinement.

### Enzyme kinetics

The kinetic parameters of WT AAC(3)-IIIa against a panel of aminoglycosides (gentamicin, kanamycin, sisomicin, tobramycin, plazomicin, neomycin, paromomycin, and ribostamycin) were obtained using the ThermoFisher NanoDrop One^C^ Spectrometer. AAC(3)-IIIa mutants were run against solely gentamicin. The acetylation of aminoglycosides was measured by a coupled assay, where the formation of pyridine-4-thiolate can be detected at 324 nm [13,14]. The kinetic assays were performed at room temperature (22°C) in quartz cuvettes (pathlength 1 cm) at a final volume of 0.8 ml. Reaction solution contained 25 mM MOPS pH 6.5, 100 mM NaCl, 500 μM 4,4’-dipyridyl disulfide (Aldrithiol™-4, Sigma-Aldrich), 150 μM acetyl coenzyme A, and aminoglycoside concentrations varying from 1.25 to 160 μM. The reaction was initialized by the addition of AAC(3)-IIIa (WT and mutants) to a final concentration of 0.3 to 0.5 μM, where UV absorbance was measured over 5 minutes. All reactions were run in triplicate, and data analysis was performed using the GraphPad5 software.

## Results and discussion

### Overall fold and substrate binding to AAC(3)-IIIa

**Fig 1:**
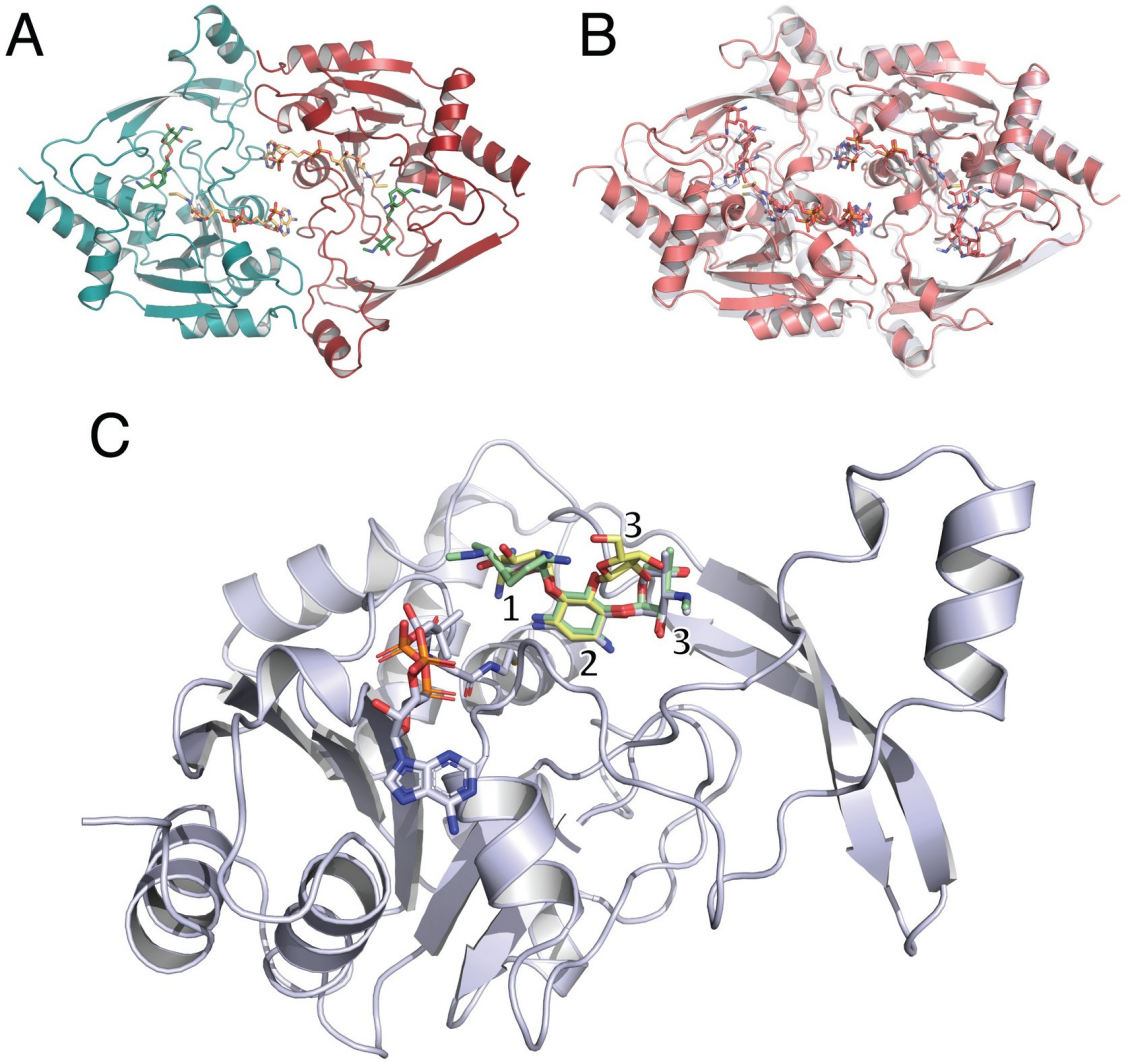
Panel A represents a dimeric structure of AAC(3)-IIIa in complex with gentamicin and CoASH. Panel B represents a structural alignment of AAC(3)-IIIa in transparent grey-blue on top of the AAC(3)-IIIb isozyme (transparent pink) bound to neomycin and CoASH (PDB ID: 6MB9), high degree of fold similarity is visible. Panel C represents a monomeric unit in ternary complex with CoASH and aminoglycosides. Neomycin is displayed in yellow, sisomicin in gray and gentamicin in green. The numbering of aminoglycosides is represented as such: 1 is the prime (′) ring, 2 is the central 2-deoxystreptamine (2-DOS) ring and 3 is the double prime (″) ring.

Three structures of AAC(3)-IIIa were solved in complex with CoASH and varying aminoglycosides, i.e., neomycin, sisomicin, and gentamicin (Fig 1). These structures are allowing us to determine the overall function of the A isozyme of the AAC(3)-III subclass of enzymes (iso^A^). Moreover, the B isozyme (iso^B^) structure has recently been solved, allowing us to compare the characteristics of enzymes belonging to this group [15]. Overall, these two isozymes have a near-identical fold (pairwise RMSD = 2.5Å) and a 58.0/44.5% sequence similarity/identity, respectively. The iso^A^ crystalizes as a homodimer, with four molecules in the asymmetric unit. This is in agreement with previously published work on iso^B^, showing its ability to exist as a homodimer in solution and *in crystallo* [15].

**Fig 2:**
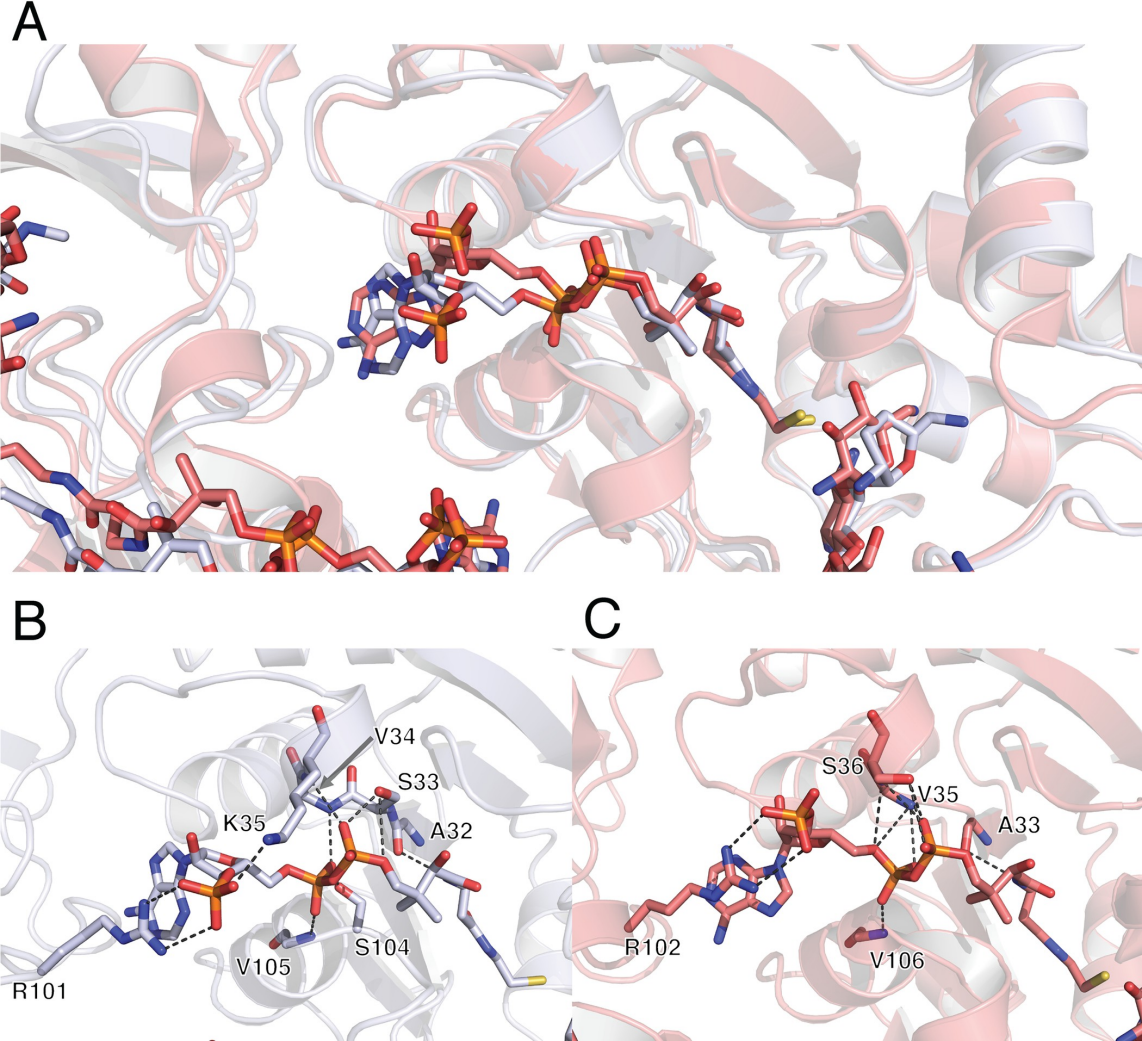
Comparison of CoASH binding to AAC(3)-IIIa and AAC(3)-IIIb. Panel A represent overall superimposition of the two structures. Panel B depicts direct interactions between sisomicin-bound AAC(3)-IIIa and CoASH, whereas panel C depicts direct interactions between neomycin-bound AAC(3)-IIIb and CoASH (PDB ID: 6MB9).

The overall conformation of CoASH in iso^A^ is stabilized by nine direct protein-ligand interactions (Fig 2). When directly comparing the binding of CoASH to iso^A^ and iso^B^, the overall mode is highly conserved, while the precise CoASH interactions vary in their number and chemistry. The main difference between CoASH binding for the two isozymes is observed in the adenosine part of the CoASH, where this portion of the molecule flips approximately 180˚ from its counterpart in iso^B^ (Fig 2). In iso^B^ the positioning of the adenine head of CoASH is due to direct interaction with the other monomer within the physiological dimer, where R102 forms a π-cation stacking with adenine moiety [15]. In iso^A^ the R101 sidechain (equivalent of R102 in iso^B^) from the other monomer within the physiological dimer is responsible for making the same π-cation stacking with an adenine head as seen in iso^B^. A potential reason for the difference in the positioning of the adenines between the two isozymes could be due to the shift of 3’-phosphate by 3.5Å due to K35 forming an interaction in iso^A^, and not iso^B^ (Fig 2). An additional reason for this flip could be explained by lack of directionality of π-cation interaction.

Iso^A^ can detoxify a broad range of 4,5- and 4,6-disubstituted aminoglycoside substrates, including tobramycin, gentamicin, dibekacin, sisomicin, kanamycin, netilmicin, paromomycin and lividomycin. These two aminoglycoside groups are built upon a two-ringed neamine core structure consisting of the central and prime rings, and an additional sugar (double-prime ring) linked to either the 5- or 6- position of the central ring, respectively. Our structures are highlighting iso^A^’s ability to bind 4,5-disubstituted aminoglycosides, i.e., neomycin, and 4,6-disubstituted aminoglycosides, i.e., sisomicin and gentamicin.

**Fig 3:**
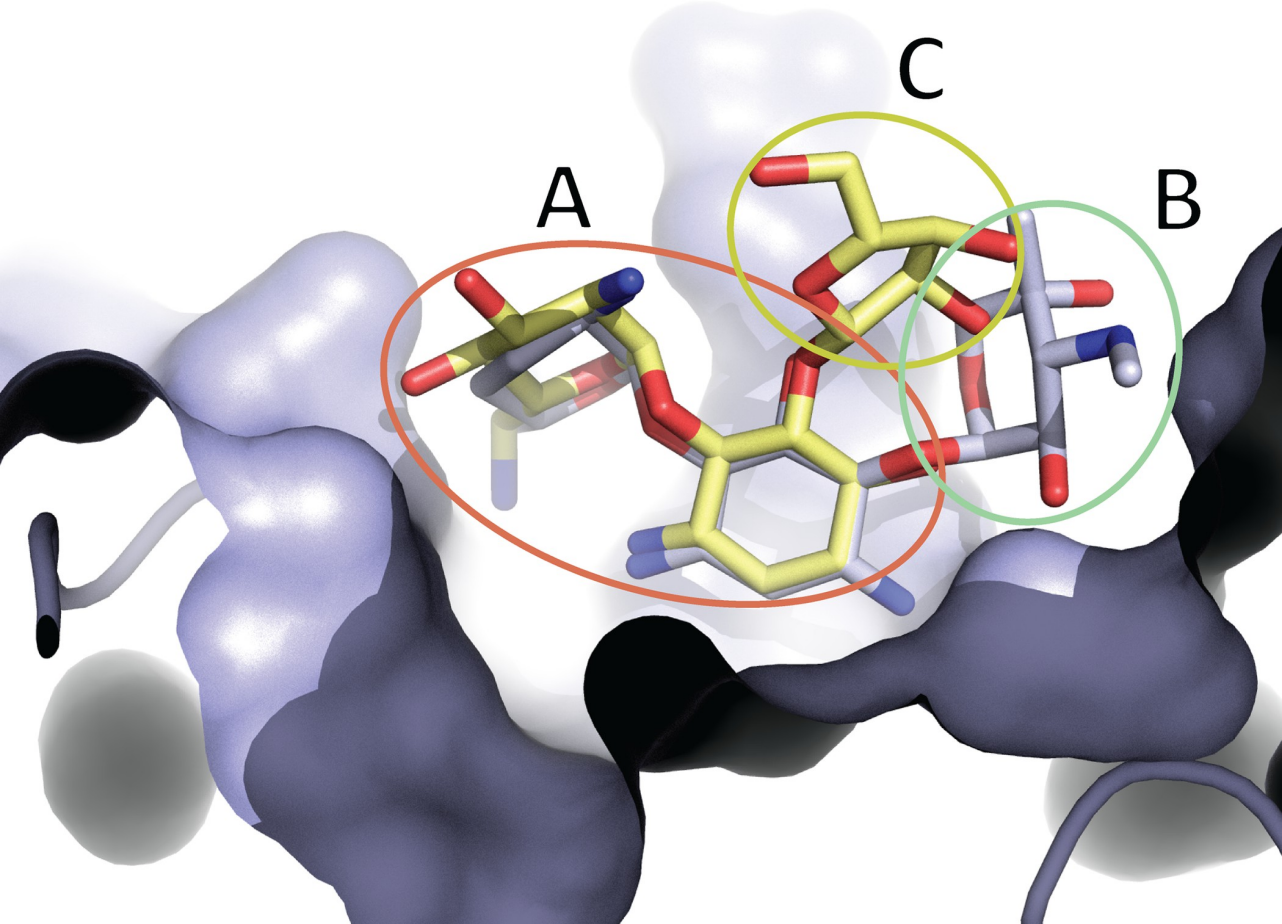
Representation of AAC(3)-IIIa’s tripartite aminoglycoside binding site, with sisomicin (blue) and neomycin (yellow). Subsite A encompasses central and prime ring of both aminoglycosides shown. Subsite B and C bind double prime ring of 4,6- and 4,5- disubstituted aminoglycosides, respectively.

The substrate promiscuity of iso^A^ is due to its tripartite active site, which can accommodate a variety of aminosugar ring attachments, this architecture was first described in APH(3’)-IIIa [16] and subsequently found in iso^B^ [15]. Briefly, the aminoglycoside binding site of iso^A^, iso^B^ and APH(3’)-IIIa can be divided into three subsites, with each being specific for a certain component of aminoglycosides. Subsite A binds the central and prime rings of 4,5- and 4,6- disubstituted aminoglycosides. Subsite B forms interactions with double prime ring of 4,6-disubstituted aminoglycosides and subsite C accommodates double prime ring of 4,5-disubstituted aminoglycosides. (Fig 3).

**Fig 4:**
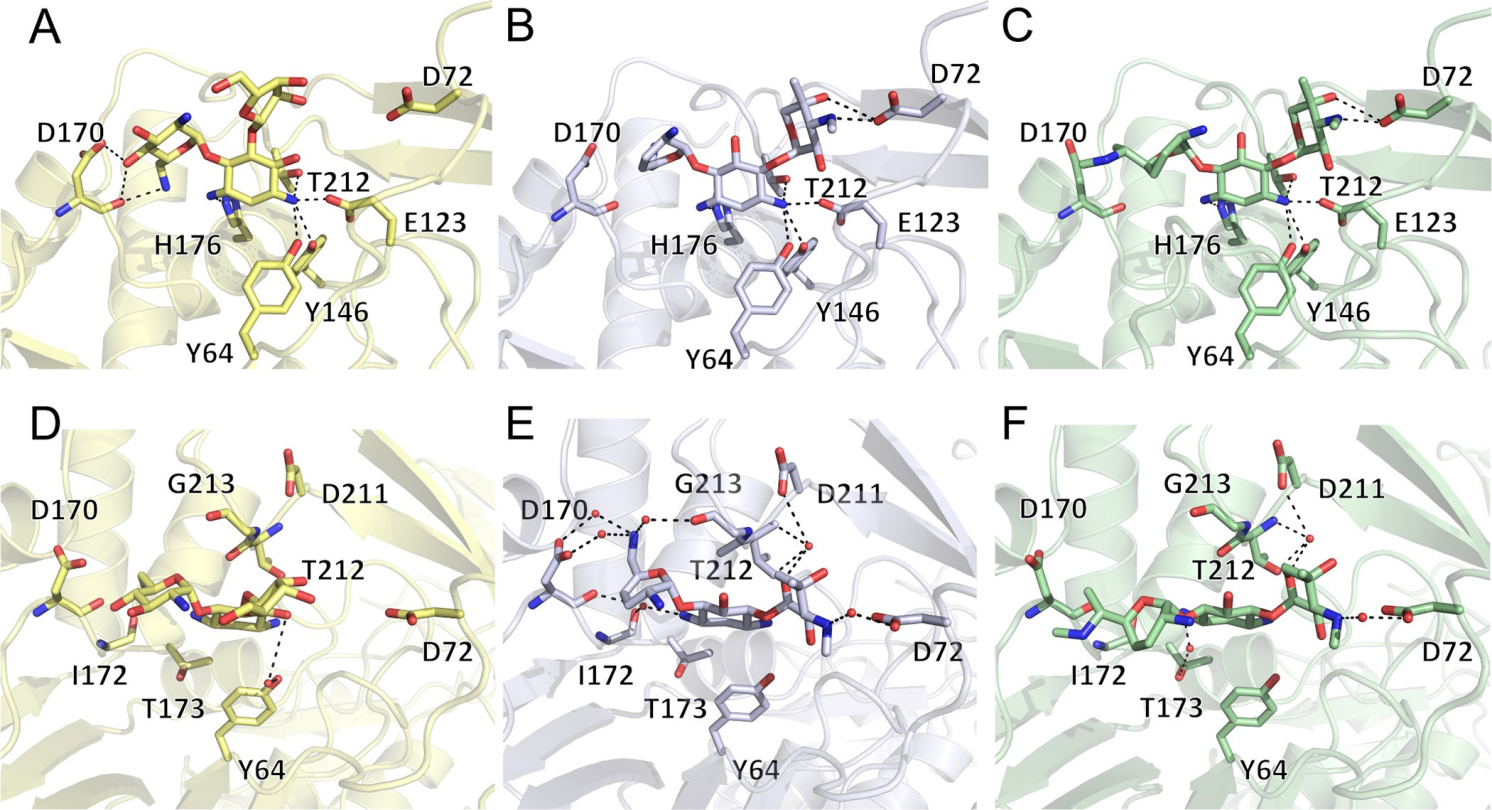
Representation of iso^A^ binding to neomycin, sisomicin and gentamicin, in yellow, silver, and green, respectively. Panels A, B and C show direct hydrogen-bond interactions between the enzyme and aminoglycosides. Panels D, E and F show structures that have been rotated 90°, to showcase water-mediated interactions between the three aminoglycosides and enzyme.

The binding of an antibiotic is fulfilled by a network of direct and water-mediated enzyme-aminoglycoside interactions. The main source of direct interactions occurs at the common central ring, where all three aminoglycoside-bound structures show that neomycin, sisomicin, and gentamicin are heavily stabilized at the N-1 position through interactions with Y64, E123, Y146, and T212. Also, the N-3 position makes a hydrogen bond with H176, which is conserved among AAC(3) enzymes and has been implicated in catalysis [17] (Fig 4).

Focusing on the structural basis for 4,5-disubstituted aminoglycoside binding, our neomycin-bound structure depicts that this antibiotic has few direct and water-mediated interactions with the enzyme. In addition to the previously mentioned central ring interactions, the enzyme also interacts with the neamine moiety of neomycin at the prime ring. The sidechain and mainchain of D170 forms interactions with the 2′-amine and 3′-hydroxyl groups. Additionally, a water-mediated interaction between the enzyme and antibiotic occurs between Y64 and N-1 of the central ring (in this model, Y64 can interact directly or indirectly with the neomycin). Finally, the subsite C contains the double-prime ring of neomycin, where linkage at the 5-position of the central ring directs the double-prime part of neomycin into a solvent-exposed region. Furthermore, even though the double-prime ring does not form any direct or water-mediated interactions, it is adequately accommodated by the enzyme’s subsite C. At this point, it is also worth mentioning that due to poor electron density, the triple-prime ring of neomycin was not modelled.

Our structures of iso^A^ bound to sisomicin and gentamicin are also allowing us to outline this enzyme’s specificity towards 4,6-disubstituted aminoglycosides. For sisomicin, the prime ring binds to three water molecules which can coordinate the sidechain of D170 and backbone ketone of G213. Unlike the double-prime ring for neomycin, sisomicin’s double prime ring is stabilized by a mix of water-mediated and direct interactions. D72 can form direct interactions with the 4″ hydroxyl and 3″ amine, while it is also responsible for a water-mediated interaction with the 3″ amine. A second water molecule mediates interactions between O-6″ and D211 and T212. Sisomicin interacts with one final water molecule at the N-3 position of the central ring, which is coordinated by the backbone atoms of D170 and I172.

Our second 4,6-disubstituted aminoglycoside-bound structure features gentamicin in the active site. Gentamicin presents a similar binding pattern to sisomicin, especially at the double prime ring, which could be attributed to the identical architecture of central and double-prime ring of those two antibiotics. Of notable difference is the complete lack of interactions between the prime ring of gentamicin and the enzyme. This could potentially be due to gentamicin not being able to form the same water-mediated interactions as sisomicin at the 6’ position (sisomicin contains a primary amine moiety whereas gentamicin contains a secondary amine). Lastly, gentamicin forms one additional interaction with the sidechain of T173, which forms a water-mediated interaction with the central ring at the N-3 position.

**Fig 5:**
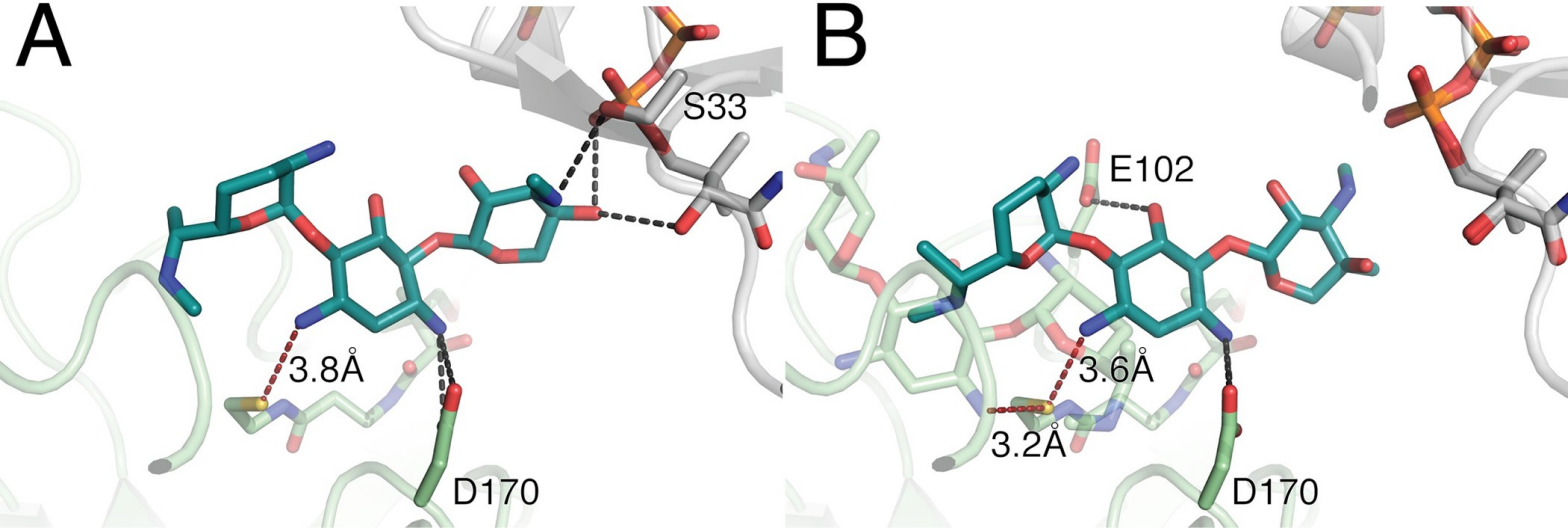
Interactions between AAC(3)-IIIa and gentamicin. Alternative binding conformation is shown in teal, and the symmetry mate in gray. Panel A represents the binding of gentamicin in chain A, where only one molecule of aminoglycoside was modeled. Panel B represents binding of gentamicin in chain B, where two molecules were alternative, and main (semi-transparent green) binders were modeled in. Hydrogen bonds between alternative mode of gentamicin binding and AAC(3)-IIIa are displayed in black, whereas distances between N-3 of the central ring are shown in burgundy.

**Fig 6:**
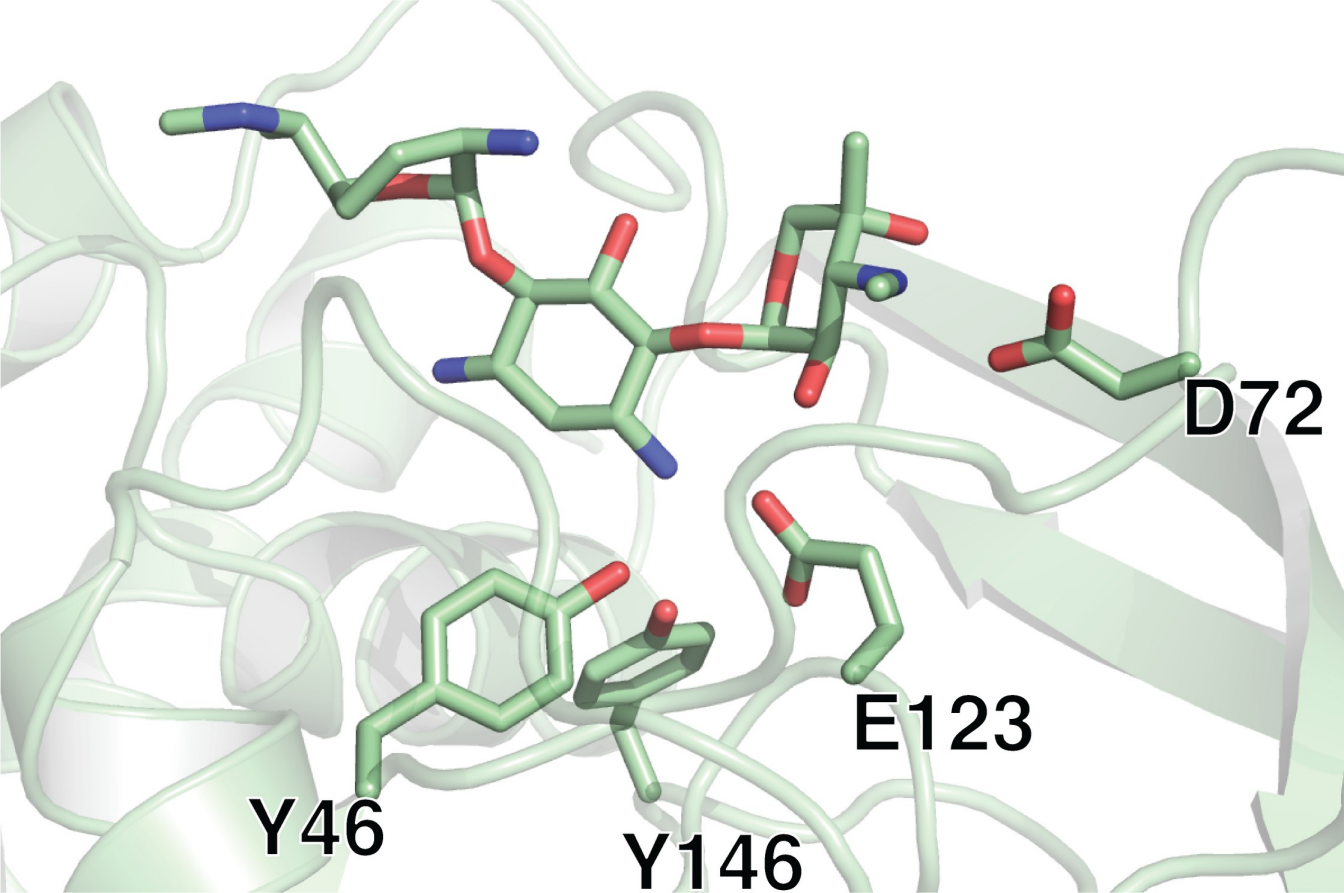
Residues implied in binding of gentamicin to the main aminoglycoside binding site.

Upon further examination of the gentamicin-bound structure of iso^A^, two of the four monomers in the unit cell revealed a different binding mode for gentamicin as that described above. In comparison to the gentamicin bound to the primary site, the aminoglycoside is rotated 180° and translated in such a manner that the N-3 of the central ring is 3.6–3.8 Å away from the terminal sulfur of the CoASH, potentially placing it in a position where the enzyme could acetylate this position (Fig 5). To determine the physiological relevance of the secondary binding site, several mutants were designed to interrupt both binding sites of gentamicin (Figs 5 and 6). Residue D170 was chosen for mutagenesis as it was the only amino acid with consistent involvement in gentamicin’s alternative binding site. Whereas Y64, D72, E123 and Y146 were selected for mutagenesis to disrupt the canonical binding site of aminoglycosides.

Several mutants were able to retain the catalytic activity including D170F responsible for the alternative binding site. Additionally, three mutants (Y64F, D72W and Y146F) targeting the main binding site retained catalytic activity. Mutants Y64F and Y146F removed one hydrogen bond at a time, which might not have been sufficient to disrupt the enzyme-substrate complex formation. D72W mutant being catalytically active could be attributed to the nature of double-prime ring, whereby this ring could adopt a different conformer, retaining the ability to bind to the active site. However, the E123F mutant has completely abolished the enzymatic activity, which could be due to two factors. In the first instance the chemistry of the interaction has changed from a charged, to hydrophobic residue. The second important factor stems from the additional volume that phenylalanine exhibits as compared to glutamic acid. This steric hindrance between N-1 of central ring might not allow for the correct positioning of aminoglycoside, resulting in catalytically dead enzyme (Table 3).

**Table 3:** Enzymatic assay results between AAC(3)-IIIa mutants and gentamicin.

	k_cat_ (s^-1^)	K_m_ (μM)	k_cat_/K_m_ (s^-1^·μM)
WT	6.12 ± 0.25	7.1 ± 1.1	0.86 ± 0.17
Y64F	12.5 ± 0.5	14.3 ± 1.7	0.87 ± 0.14
D72W	3.59 ± 0.14	19 ± 2	0.19 ± 0.03
E123F	N/A	N/A	N/A
Y146F	4.33 ± 0.20	8.5 ± 1.5	0.51 ± 0.11
D170F	3.40 ± 0.11	4.7 ± 0.6	0.72 ± 0.12

This result led us to the conclusion that while the primary binding site of aminoglycosides can be disrupted to the point of total enzymatic inactivity, the alternative site seems to carry no physiological relevance and is speculated to be merely a crystallographic artifact. This is also consistent with a proposed role of H176 in catalysis, as in the second binding mode this residue is not in close proximity to N-3 of gentamicin [17].

Additional kinetic analyses of WT iso^A^ tested the enzyme’s ability to modify a large array of aminoglycosides from both groups, including 4,5-disubstituted neomycin, paromomycin, and ribostamycin, and 4,6-disubstituted sisomicin, gentamicin, tobramycin, kanamycin, and plazomicin (Table 4). Based on our analysis, iso^A^ does not preferentially detoxify one group over the other. The catalytic efficiency demonstrates that while sisomicin and kanamycin are both 4,6-disubstuted antibiotics, they are the most and least efficiently converted substrates, respectively. Interestingly, although K_cat_ and K_m_ values vary for the different substrates, the enzyme efficiency stays within the narrow range of 0.6–1.0 s^-1^·μM, where poor K_cat_ gets compensated by more favourable K_m_ and vice-versa. Our analysis also includes the newest next-generation aminoglycoside plazomicin. Plazomicin is a semi-synthetic derivative of gentamicin and includes a hydroxyethyl extension at the N-6’ position and a (*s*)-4-amino-2-hydroxybutarate (HABA) group at the N-1 position. Other aminoglycosides, such as butirosin and amikacin, which also introduce a HABA tail at the N-1 position of their chemical structure, are not substrates of iso^A^. Based on our crystal structures, we can deduce that the N-1 HABA substituent introduces a steric clash that prevents these aminoglycosides from binding to iso^A^. It is predicted that hydroxyethyl chain on plazomicin’s 6’ position would be able to be accommodated, as homologous 6’ amine of sisomicin is solvent exposed. Therefore, it is unsurprising that our kinetic data reveal that plazomicin is also not a substrate of iso^A^.

**Table 4:** Kinetic parameters for antibiotics.

Aminoglycoside	k_cat_ (s^-1^)	K_m_ (μM)	k_cat_/K_m_ (s^-1^·μM)
4,5-disubstituted aminoglycosides			
Neomycin	6.15 ± 0.20	6.6 ± 0.8	0.93 ± 0.14
Paromomycin	5.52 ± 0.21	6.7 ± 0.9	0.82 ± 0.14
Ribostamycin	5.38 ± 0.23	7.9 ± 1.1	0.68 ± 0.13
4,6-disubstituted aminoglycosides			
Sisomicin	10.48 ± 0.34	10.9 ± 1.2	0.97 ± 0.14
Gentamicin	8.18 ± 0.27	10.1 ± 1.2	0.81 ± 0.13
Kanamycin	4.45 ± 0.19	7.0 ± 1.1	0.63 ± 0.13
Tobramycin	5.68 ± 0.24	6.6 ± 1.1	0.86 ± 0.18
Plazomicin	N/A	N/A	N/A

## Conclusions

AAC(3)-IIIa and its fellow isozymes utilize both 4,5- and 4,6-disubstituted aminoglycosides providing this enzyme with the broadest substrate specificities of any AAC(7). This broad spectrum of specificities can be attributed to the tripartite architecture of the aminoglycoside binding site, similar to both AAC(3)-IIIb [15] and APH(3’)-IIIa [16]. The blend of direct and water-mediated interactions found in the A and B subsites accept the neamine core and the double prime ring of 4,6-disubstituted aminoglycosides. Furthermore, we found that subsite C lacks any direct or water-mediated interactions; but it does provide space to house the double and triple prime rings of 4,5-aminoglycosides that protrude from the O-5 of the central ring. Due to the openness of the aminoglycoside binding site, together with crystal packing, an alternative binding mode of gentamicin was found. In this case, AAC(3)-IIIa formed direct interactions with the antibiotic in two, out of the four monomers solved. But based on mutagenesis data this phenomenon has been designated as a crystallographic artifact. AAC(3)-IIIa has a very broad spectrum of specificities towards varied aminoglycosides and we found that it utilizes 4,5- and 4,6-disubstituted aminoglycosides non-preferentially, where the iso^A^ displays similar enzymatic efficiency despite the differences in K_cat_ and K_m_. Out of the panel of antibiotics tested, plazomicin, the next generation aminoglycoside, was not utilized as a substrate of this enzyme. This is explained by the structural data as any substituents at the N-1 of the central ring cannot be accommodated by this protein.

The findings reported here should prove helpful in identifying and developing solutions for addressing aminoglycoside antibiotic resistance. Specifically, two avenues can be envisioned, guided by the structural data presented. First, inhibitors of AAC(3) enzymes can be developed, as has been done for AAC(6’)-Ii [18], for use as antibiotic adjuvants. Perhaps, it might be feasible to target multiple AAC families, to increase the clinical utility, analogs to efforts pursued for kinases that confer aminoglycoside resistance [19]. Secondly, the detailed structural data on how AAC(3)-IIIa binds various aminoglycosides can inform the development of next-generation aminoglycosides that are resilient to enzymatic modification by a plethora of AGMEs [20,21].
